# Coronary Perfusion After Valve-in-Valve Transcatheter Aortic Valve Implantation in Small Aortic Root: In Vitro Experimental Assessment

**DOI:** 10.1007/s12265-023-10364-y

**Published:** 2023-04-25

**Authors:** Michal Jaworek, Guido Gelpi, Francesca Perico, Claudia Romagnoni, Giordano Tasca, Eleonora Salurso, Monica Contino, Alberto Redaelli, Gianfranco Beniamino Fiore, Riccardo Vismara

**Affiliations:** 1 Department of Electronics, Information and Bioengineering, Politecnico Di Milano, Via Golgi 39, 20133 Milan, Italy; 2 ForcardioLab—Fondazione per la Ricerca in Cardiochirurgia ONLUS, Milan, Italy; 3 Cardiac Surgery Unit, Fondazione IRCCS Ca’ Grande Ospedale Maggiore Policlinico, Milan, Italy; 4 Cardiac Surgery Department, Heart Health Center, King Saud Medical City, Riyadh, Kingdom of Saudi Arabia

**Keywords:** Valve-in-valve, TAVR, TAVI, Aortic valve, Coronary flow, Coronary obstruction, Aortic root, In vitro, Bench

## Abstract

Coronary flow obstruction following transcatheter aortic valve-in-valve implantation (VIV-TAVI) is associated with a high mortality risk. The aim of this work was to quantify the coronary perfusion after VIV-TAVI in a high-risk aortic root anatomy. 3D printed models of small aortic root were used to simulate the implantation of a TAVI prosthesis (Portico 23) into surgical prostheses (Trifecta 19 and 21). The aortic root models were tested in a pulsatile in vitro bench setup with a coronary perfusion simulator. The tests were performed at baseline and post-VIV-TAVI procedure in aligned and misaligned commissural configurations under simulated hemodynamic rest and exercise conditions. The experimental design provided highly controllable and repeatable flow and pressure conditions. The left and right coronary mean flow did not differ significantly at pre- and post-VIV-TAVI procedure in any tested configurations. The commissural misalignment did not induce any significant alterations to the coronary flow.

**Graphical Abstract:**

High-risk aortic root anatomy did not trigger coronary ostia obstruction or coronary flow alteration after transcatheter aortic valve implantation in a surgical bioprosthesis as shown from in-vitro flow loop tests.

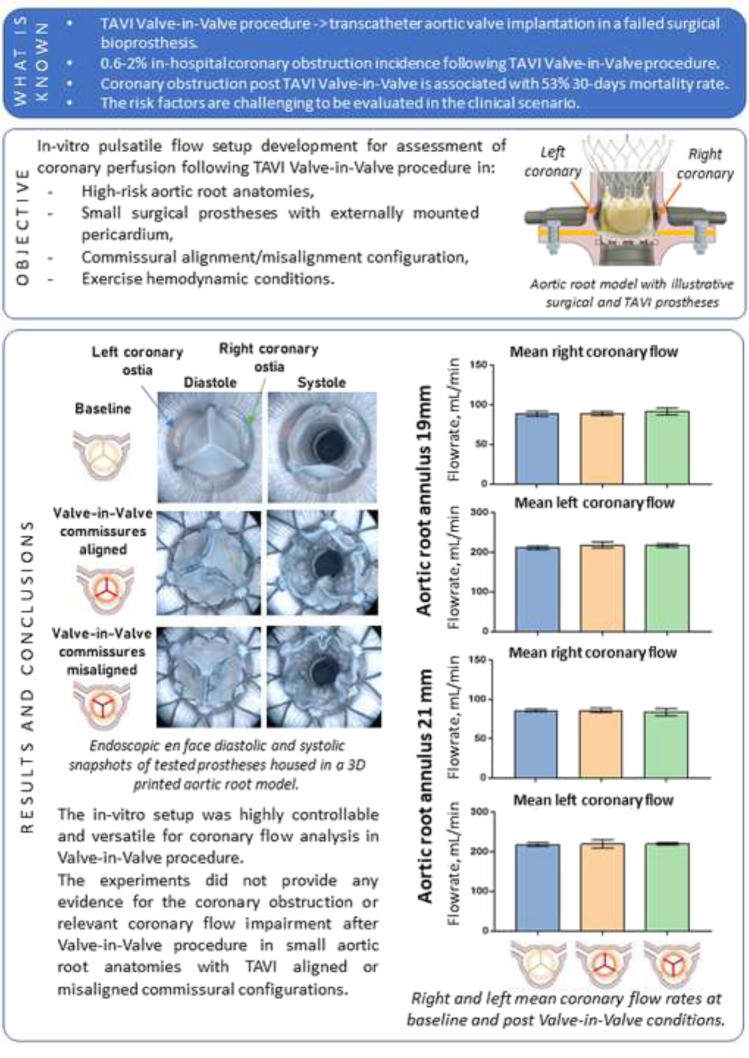

**Supplementary Information:**

The online version contains supplementary material available at 10.1007/s12265-023-10364-y.

## Introduction

Transcatheter aortic valve-in-valve implantation (VIV-TAVI) is, currently, a IIa class recommendation for surgical aortic valve bioprosthesis (SAVB) failure [1]. Supported by growing scientific evidence [2], the Heart Team chooses VIV-TAVI for degenerated SAVB depending on anatomic considerations, SAVB design features, and patient characteristics. In large multicenter studies of VIV-TAVI in high-risk patients, 30-day mortality can range from 2.2 to 2.9%; in-hospital coronary obstruction rates vary from 0.6 to 2% and the conversion rate to open heart surgery is very low (0.2%) [3]. The coronary flow obstruction or disruption is related to the interaction between the SAVB leaflets, the TAVI, and the aortic root (AR) and it is one of the riskiest complications following VIV-TAVI, associated with 52.9% 30-day mortality rate [4].

Coronary ostia obstruction rate has been reported to be 4- to sixfold higher in VIV-TAVI than what has been reported for TAVI in native aortic valve, particularly for SAVB with externally mounted leaflets [5]. In VIVID, the largest VIV-TAVI registry, an increased mortality (nearly 25%) was observed among patients with small size SAVB [4].

Some morphometrical features of the AR, namely the sinotubular junction (STJ) diameter, the sinus of Valsalva diameter (SOVD), the coronary ostia height, and the valve-to-coronary distance (VTCD), have been identified clinically as factors associated to the impairment of coronary flow after VIV-TAVI [4]. Narrow ARs, already implanted with SAVB, may have limited space for the SAVB’s dislodged leaflets, potentially compromising coronary ostia flow. SAVB leaflets’ height can also interfere with coronary ostia flow. In particular, a left VTCD between 6 and 3 mm and a coronary height lower than 10 mm have been recognized as a high-risk anatomy for coronary occlusion [6]. Jabbour and colleagues reported that 59.3% of all coronary occlusion events occurred in patients with a ≤ 3-mm difference between mean SOVD and SAVB size implanted [7].

In order to preserve coronary ostia access and consequently an adequate coronary flow, it has been postulated that keeping an alignment of the transcatheter valve commissures with respect to the native aortic valve commissures is of paramount importance [8].

Coronary occlusion is associated with very high mortality rates, but coronary flow after VIV-TAVI has been rarely investigated and the underlying mechanisms are poorly understood. Clinically, the phenomenon is complex to be studied due to multiple confounding factors. There is a need for a highly controllable experimental approach that enables in-depth studies of coronary occlusion or alteration phenomena in VIV-TAVI.

The aim of this work is the development of a high reproducible bench model and an experimental protocol for the evaluation of VIV-TAVI performance able to quantify the coronary perfusion after VIV-TAVI in a high-risk AR configuration. The tests were performed under rest and exercise conditions to investigate any possible coronary flow difference induced by stress conditions.

## Methods

### Valve-in-Valve Model

**Fig. 1:**
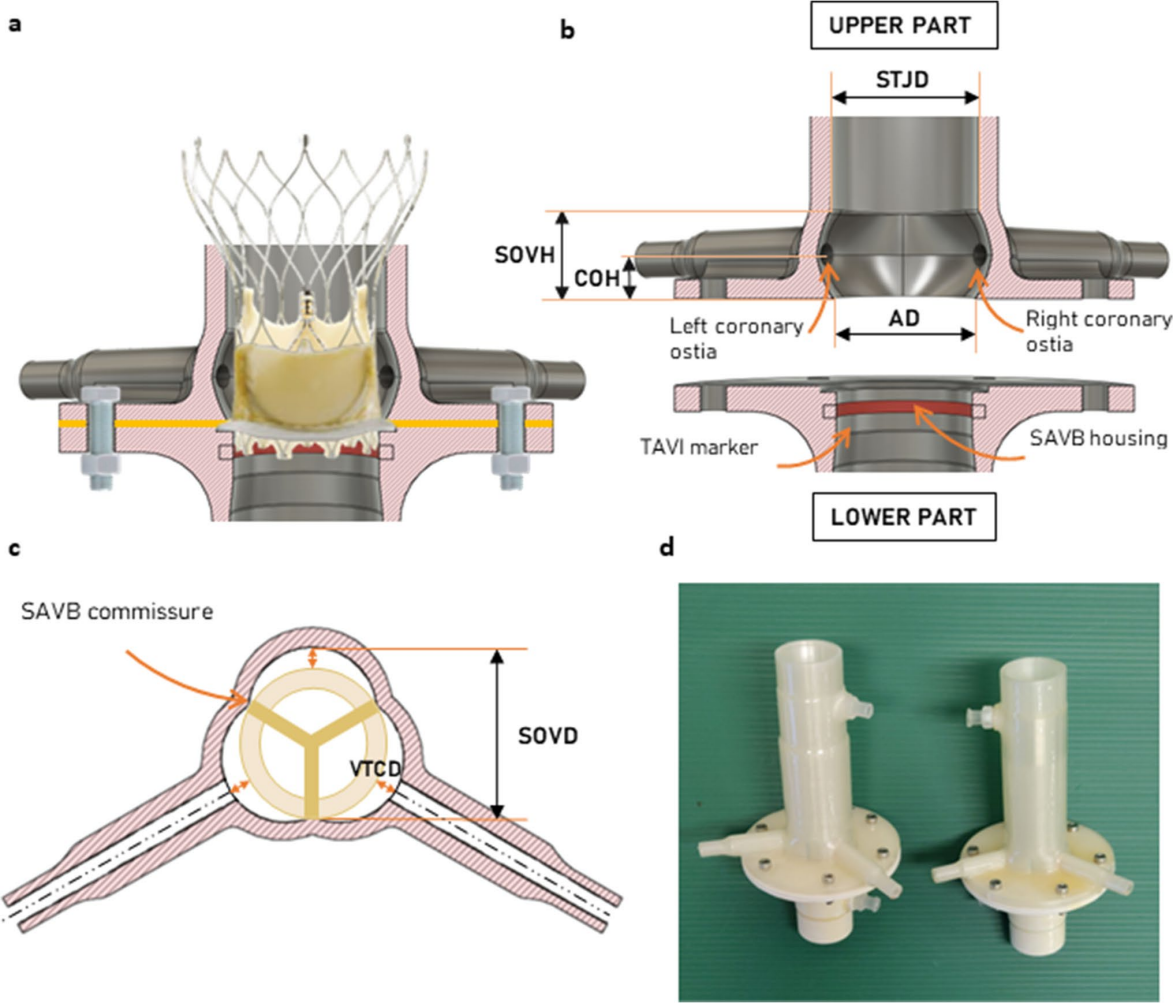
**a** Long-axis section of the aortic root (AR) model with a schematic view TAVI and SAVB placed inside the model. **b** Long-axis section of the AR model divided into two parts and the significant geometric parameters are reported. AD, diameter of the annulus; SOVH, height of the sinus of Valsalva; STJD, diameter of the sinotubular junction; COH, height of coronary ostia. **c** Short-axis section of the AR model at the level of coronaries with SAVB model (yellow circular crown). SOVD, diameter of the sinus of Valsalva; VTCD, valve-to-coronary distance. **d** 3D printed models of the AR

A self-expandable TAVI prosthesis (Portico Transcatheter Heart Valve, St. Jude Medical, St. Paul, MN, USA) with label size 23 (2 samples) was implanted in a SAVB with externally mounted leaflets (St. Jude Trifecta Valve with Glide Technology (GT)) of label size 19 (3 samples) or 21 (3 samples), inside a rigid, 3D-printed AR model, as shown schematically in Fig. 1a.

The Trifecta GT 19 was housed inside an AR model with aortic annulus diameter (AD) of 19 mm, while the Trifecta GT 21 was housed inside an AR model with AD of 21 mm; such AR models will be referred to as AR model 19 and AR model 21, respectively. Both AR models were made of an upper and lower part to ease SAVB mounting. The SAVB suturing ring was placed in a housing (Fig. 1b, SAVB housing) and was compressed after the model was closed exploiting a flange. The lower part had a marker on its internal wall (Fig. 1b, TAVI marker) to guide the implantation of the TAVI prosthesis 3 mm below the SAVB metallic frame, as done in the clinical practice to increase the effective orifice area and reduce post-procedural pressure difference [9, 10].

The AR models had a paradigmatic geometry, as reported in Table 1 and shown in Fig. 1b and c, featuring a symmetric design with three sinuses of Valsalva, two centered coronary ostia with an internal diameter of 3.5 mm, and a circular STJ, whose diameter was 2 mm larger than the AD. To replicate a high-risk anatomy for coronary obstruction, the valve-to-coronary distance (VTCD) was set to 4 mm [4, 6]. VTCD was defined as the distance between the SAVB wall, approximated as a cylinder of a diameter equal to the AD, and the center of the coronary ostia. The height of the sinuses of Valsalva was defined to be 2 mm higher than the SAVB height [11]. Figure 1d shows the 3D printed model of the AR.

**Table 1:** Geometrical parameters of the aortic root models

	Aortic root model 19	Aortic root model 21
Annulus diameter (AD), mm	19	21
Sinotubular junction diameter (STJD), mm	AD + 2	
Sinus of Valsalva height (SOVH), mm	12.5	13.5
Coronary ostia height (COH), mm	SOVH/2	
Coronary ostia diameter, mm	3.5	
Valve-to-coronary distance (VTCD), mm	4	
Sinus of Valsalva diameter (SOVD), mm	23.2	25.2

**Fig. 2:**
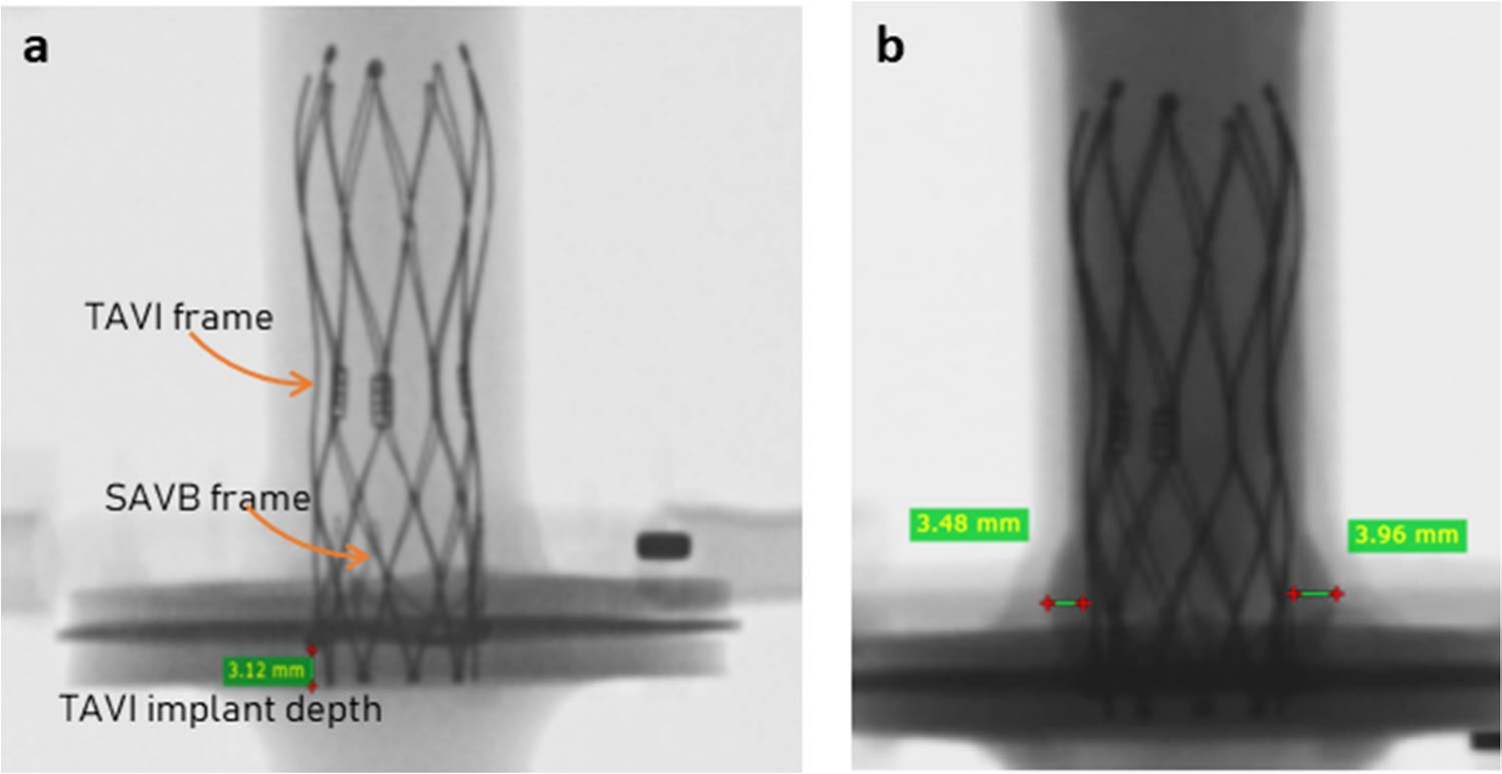
Fluoroscopic view of the Portico 23 implanted inside the Trifecta GT 19 and housed in the aortic root model 19. **a** Verification of the TAVI implantation height. **b** Verification of the valve-to-coronary distance by filling the model with contrast medium

Preliminarily, we confirmed under fluoroscopy imaging that the applied TAVI implantation strategy, exploiting the marker on the AR model internal wall, was adequate to obtain desired TAVI implantation depth, as the SAVB metallic frame was not visible in direct vision (Fig. 2a). Moreover, the AR model was filled with contrast medium and the VTCD was measured (Fig. 2b).

### Test Setup

**Fig. 3:**
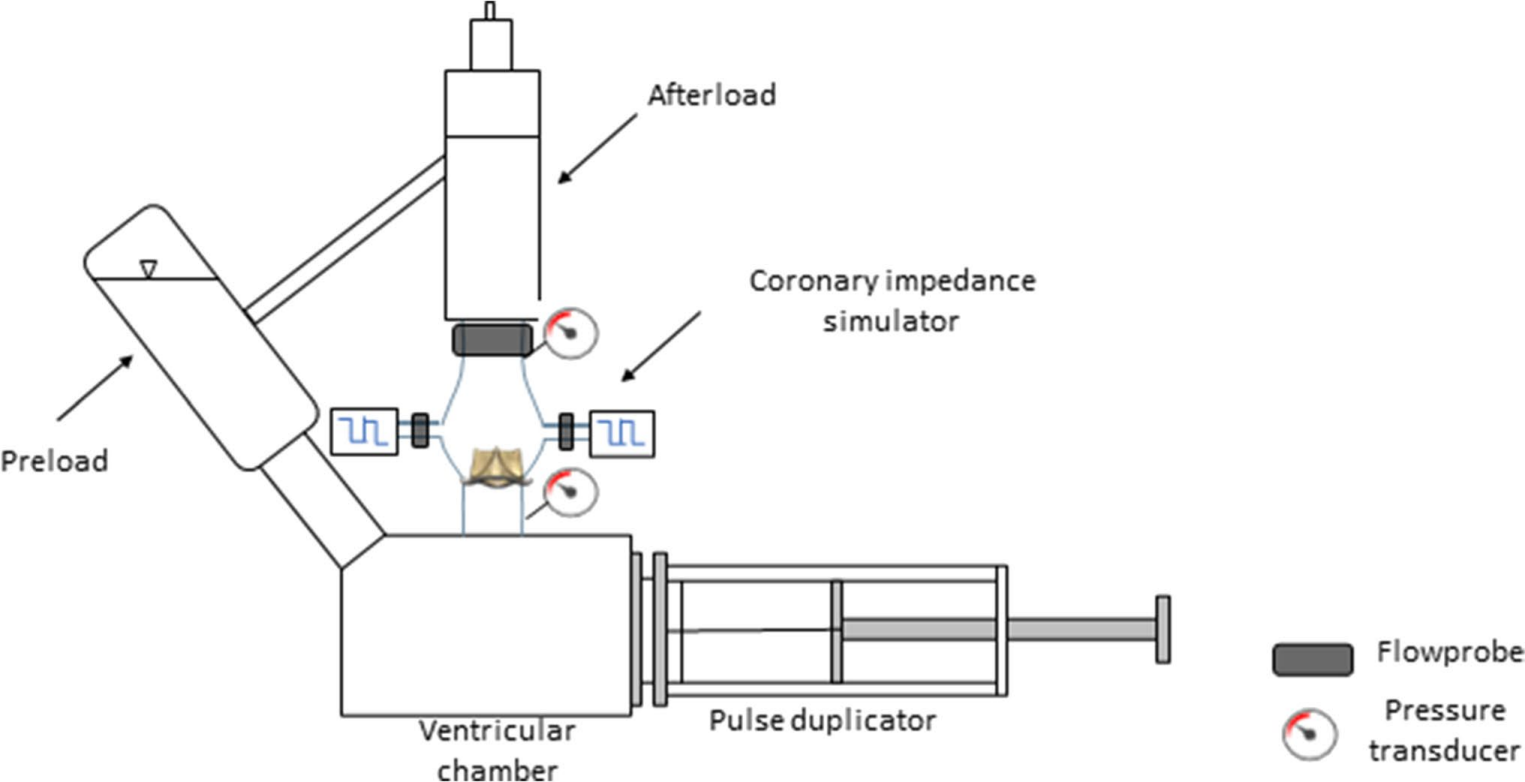
A scheme of test setup with a pulse duplicator

The AR models were incorporated into a pulsatile flow in vitro pulse duplicator [12] equipped with a coronary perfusion simulator [13] shown in Fig. 3.

This pulsatile flow system was already adopted for various experimental scenarios and used to test and develop implantable cardiac devices [14–16] or study the coronary perfusion [17]. The system allows replicating the realistic working conditions of the prosthesis. The pulse duplicator [12] consists of a custom-made pulsatile pump (motor: MCS06C41, Lenze, Hameln, Germany; controller: Servo9322EK, Lenze; software: Global Drive Control 4.14, Lenze), a rigid left ventricle chamber with a service mitral valve connected to a preload, free-surface reservoir. The AR model inflow and outflow sides were connected to the ventricle chamber outflow and to an adjustable systemic impedance simulator (afterload), respectively. The circuit was filled with saline solution under controllable 37 °C temperature.

The coronary perfusion simulator was made of two independent hydraulic circuits, designed to replicate the impedances of the left and right coronary circulations. Their inflows were connected to the left and right coronary ostia of the AR model and the outflows were connected to the preload reservoir, representing a quasi-atmospheric pressure environment. To simulate the time-variable systolic/diastolic resistance of the coronary circulation, each coronary circuit forks into two branches with independently adjustable resistances; computer-controlled pinch valves, synchronized with the pump piston velocity signal and aortic flow signal, were used to drive the flow through the systolic or diastolic resistance branches, according to the current cardiac phase.

Flow signals were measured with transit-time flowmeters. The aortic flowrate was measured with HT110R (Transonic System, Inc., Ithaca, NY, USA) equipped with a clamp-on 1″ probe and the coronary flowrates were measured with TS410 (Transonic System, Inc., Ithaca, NY, USA) equipped with two in-line 4PXN probes. Aortic pressure was measured by a piezoresistive transducer (143PC05D model, 140PC series, Honeywell, Inc., Morristown, NJ, USA). All signals were acquired with an A/D converter (DAQ USB 6210, National Instruments, Austin, TX, USA) at a sampling frequency of 200 Hz. A fiberscope (ENF-GP, Olympus Corp., Tokyo, Japan) was used to directly inspect the kinematics of the prosthesis. The hemodynamic data was averaged over 10 cardiac cycles and the following parameters were derived: cardiac output (CO, in L/min), as mean aortic flowrate; stroke volume (SV, in mL) as the integral of the aortic flow curve during the systole; backflow volume (BV, in mL) as the integral of the aortic flow curve during the diastole; mean (cycle-averaged) aortic pressure (AoP, in mmHg). The coronary flowrate (in mL/min) was evaluated as the mean flowrate during the whole cardiac cycle, or during the systole and during the diastole for the left and right branches. The coronary flowrate parameters were expressed both as absolute values, and in percentage normalized to the baseline (SAVB-alone) mean value. The normalized coronary flowrate conceptually corresponds to the fractional flow reserve (FFR), a clinical parameter used in case of coronary stenosis, defined as the ratio between the maximum achievable myocardial blood flow and the maximum theoretical myocardial blood flow. In case of no obstructions (i.e., no stenosis), FFR is close to 100%, while FFR below 80% was associated with significant coronary stenosis [18]. Based on this clinical reference, we evaluated the post-VIV-TAVI normalized coronary flow parameter as an indicator of potential coronary flow alteration. The same approach was already applied in another VIV-TAVI experimental study [19]. A cut-off of 80% was considered a relevant coronary flow impairment.

### Test Protocol

For each SAVB size, the tests were performed at baseline condition (only SAVB) and post-VIV-TAVI procedure with aligned and misaligned commissures. Misalignment was simulated with a 60° rotation angle of the TAVI commissures with respect to the SAVB commissures. All tests were run under typical rest loading conditions (heart rate 60 bpm, SV 80 mL, AoP 100 mmHg) and under simulated exercise conditions (heart rate 90 bpm, SV 80 mL, AoP 140 mmHg). The coronary simulator resistances of each branch were adjusted at the baseline rest conditions to obtain physiological left/right diastolic/systolic mean flowrate values [20]. Each testing condition was repeated 6 times combining 3 SAVB samples of the same size and 2 TAVI samples of the same size.

### Statistical Analysis

Following normal distribution assessment using Shapiro–Wilk test, the data were presented as mean ± standard deviation. The variables were compared using repeated measures three-way ANOVA considering the treatment (baseline, post-VIV-TAVI with commissures aligned, post-VIV-TAVI with commissures misaligned), the SAVB label size (19, 21), and the loading conditions (rest, exercise) as independent factors. The Bonferroni correction was used in post hoc analysis. A *p* value of < 0.05 was assumed as statistically significant. To assess for potential relevant coronary flow impairment due to VIV-TAVI procedure, an equivalence test was carried out. The 95% confidence intervals (CI) of post-VIV-TAVI normalized mean left and right coronary flowrates were compared with the cut-off value of 80% inspired to the use of FFR when analyzing clinical data [18].

## Results

**Fig. 4:**
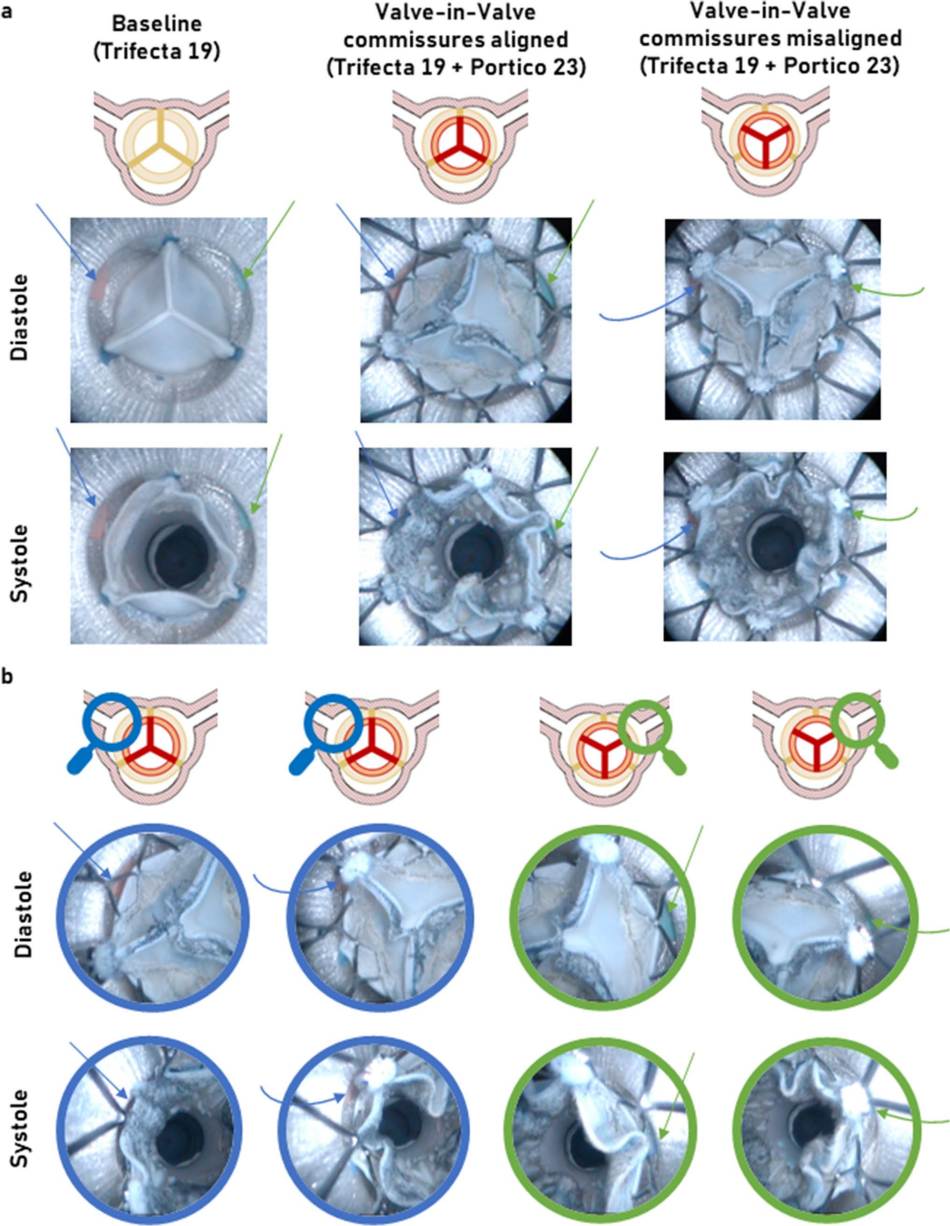
**a** Endoscopic en face diastolic and systolic snapshots of tested prostheses housed in an aortic root model of geometry considered risky for coronary occlusion at the baseline (Trifecta GT 19) and post-Valve-in-Valve TAVI treatment (Trifecta GT 19 + Portico 23) with commissural alignment and misalignment configuration. **b** Zoom-in endoscopic systolic and diastolic snapshots proximal to left (blue arrows) and right (green arrows) coronary ostia in post-Valve-in-Valve TAVI with commissural alignment and misalignment configuration

In total, 72 experimental tests were carried out. Coronary occlusion did not occur in any of the tested experimental conditions post-VIV-TAVI, regardless of the very limited space available for fluid flow between the internal wall of the AR models and the implanted prostheses. Exemplary endoscopic diastolic and systolic snapshots of the AR model 19 pre- and post-VIV-TAVI are shown in Fig. 4 and in the supplementary video (Online Resource 1).

### Working Conditions Assessment

**Fig. 5:**
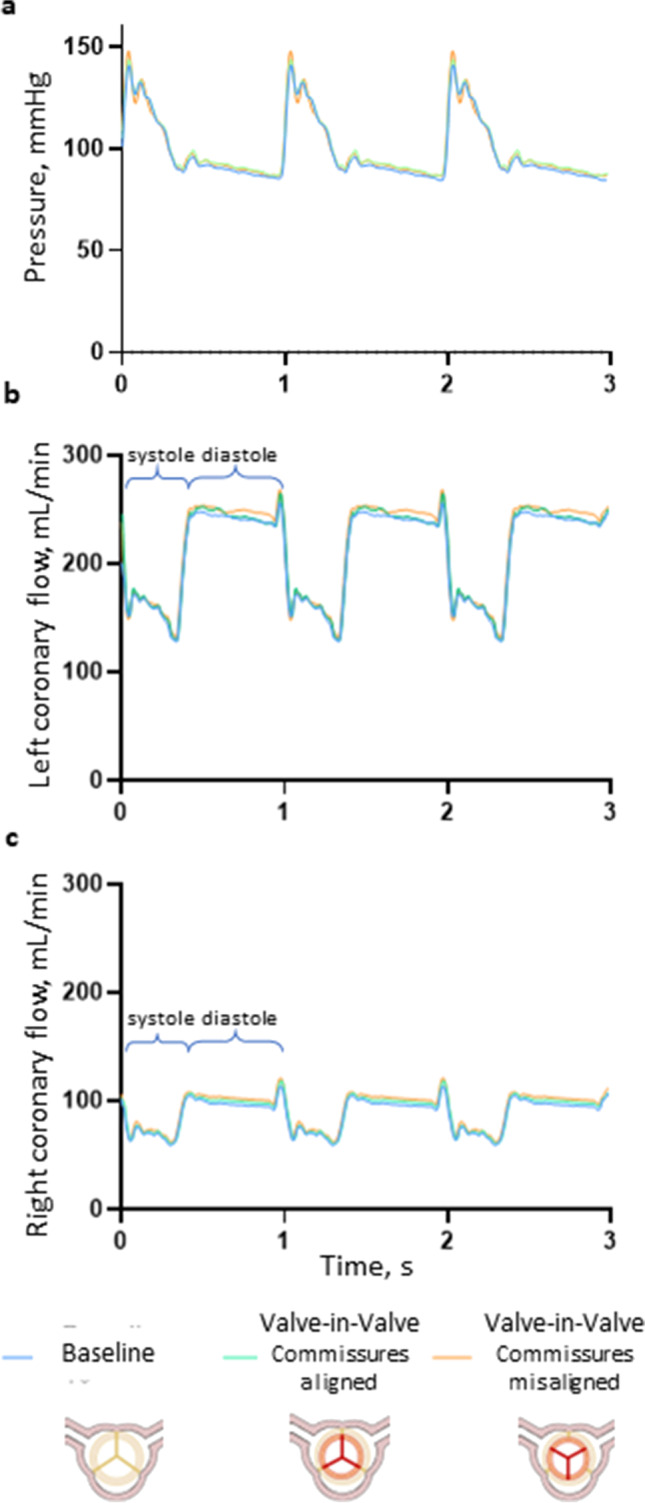
Tracings of aortic pressure (**a**), left (**b**), and right (**c**) coronary flow signals at baseline, Valve-in-Valve with commissures aligned and misaligned configuration at simulated rest conditions in aortic root model size 19

The tests were performed under controllable pressure and flow conditions (Table 2). The aortic pressure, which is the driving force of the coronary flow, was set in a very repeatable manner in terms of the mean value and the waveform (Fig. 5a). The left (Fig. 5b) and right (Fig. 5c) coronary flow waveforms were in counterphase with respect to the aortic pressure waveform, and the left and right mean coronary flowrates accounted for around 70% and 30% of total coronary flowrate, respectively, replicating in vivo physiological-like conditions [20]. The disturbance observable in coronary flow waveforms, especially during the systolic phase, reflected the aortic pressure waveform oscillations which were related to the fluid inertial effects. The flow spikes present in both left and right coronary flow waveforms were related to the fluid displacement in the coronary simulator tubing due to pinch valve switching.

**Table 2:**
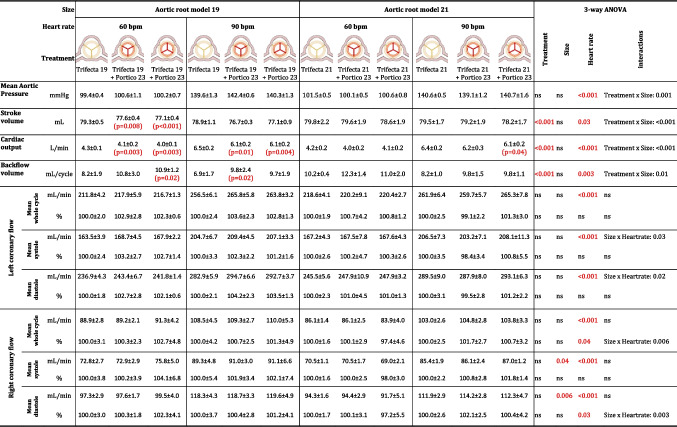
In vitro hemodynamic results of TAVI Valve-in-Valve implantation of Portico 23 in Trifecta GT 19 and Trifecta GT 21 prostheses in aligned and misaligned configurations in rest and exercise simulated conditions. The prostheses were implanted in aortic root models with geometries considered clinically as high risk for coronary occlusion. All data are presented as mean ± standard deviation (*p* value with respect to baseline for the same heart rate and aortic root model)

The maximum mean difference of AoP throughout all experimental tests was of 2.1 mmHg and 3.3 mmHg for rest and exercise conditions, respectively. Similarly, stroke volume was well controllable with maximum mean difference throughout test conditions of 2.7 mL/cycle. In case of AR model 19 at rest conditions, the stroke volume decreased significantly post-VIV-TAVI with respect to baseline conditions, which could be related to minor leakages from the circuit between the AR model and the flow probe. Nonetheless, the mean absolute differences (< 2.2 mL/cycle) can be considered negligible.

Three-way ANOVA (Table 2) revealed a significant decrease of CO post-VIV-TAVI (*p* < 0.001) which was related to the increase of BV post-VIV-TAVI. The increase of BV (max mean difference < 3 mL/cycle) could be linked to cumulative effect of transvalvular (among TAVI leaflets) and paravalvular (between TAVI skirt and SAVR leaflets) leakages in the VIV-TAVI configuration with respect to the baseline configuration.

Passing from rest to exercise conditions, a statistically significant increase of CO and AoP and decrease of BF were obtained (*p* < 0.001), as expected.

The baseline coronary flowrate was highly repeatable, and it was possible to set the typical physiological values of mean diastolic and systolic, left and right coronary flowrates. The highest coefficient of variation (i.e., the ratio between standard deviation and the mean value) of the coronary flowrate was 4%.

### Coronary Flow Assessment

In any of the performed tests, the implantation of TAVI prosthesis in SAVB did not cause coronary ostia occlusion or relevant coronary flow impairment. Three-way ANOVA (Table 2) did not yield any statistically significant changes related to the treatment (baseline, VIV-TAVI in aligned and misaligned commissures configuration) for any of the coronary flowrate parameters (whole cardiac cycle, diastole or systole in the left or in the right coronary branch). The change of the heart rate induced significant increase of all evaluated left and right coronary flowrate parameters (*p* < 0.001). The AR model size did not yield statistically significant difference in left coronary flow parameters. Significant difference of the mean systolic (*p* = 0.04) and diastolic (*p* = 0.006) right coronary flowrate related to the AR model size was detected. This was associated with the differences in the absolute values in the baseline conditions due to manual setting of the coronary simulator. In fact, when the coronary flowrate parameters were normalized to the baseline mean values, the AR model size no more resulted to induce statistically significant changes.

**Fig. 6:**
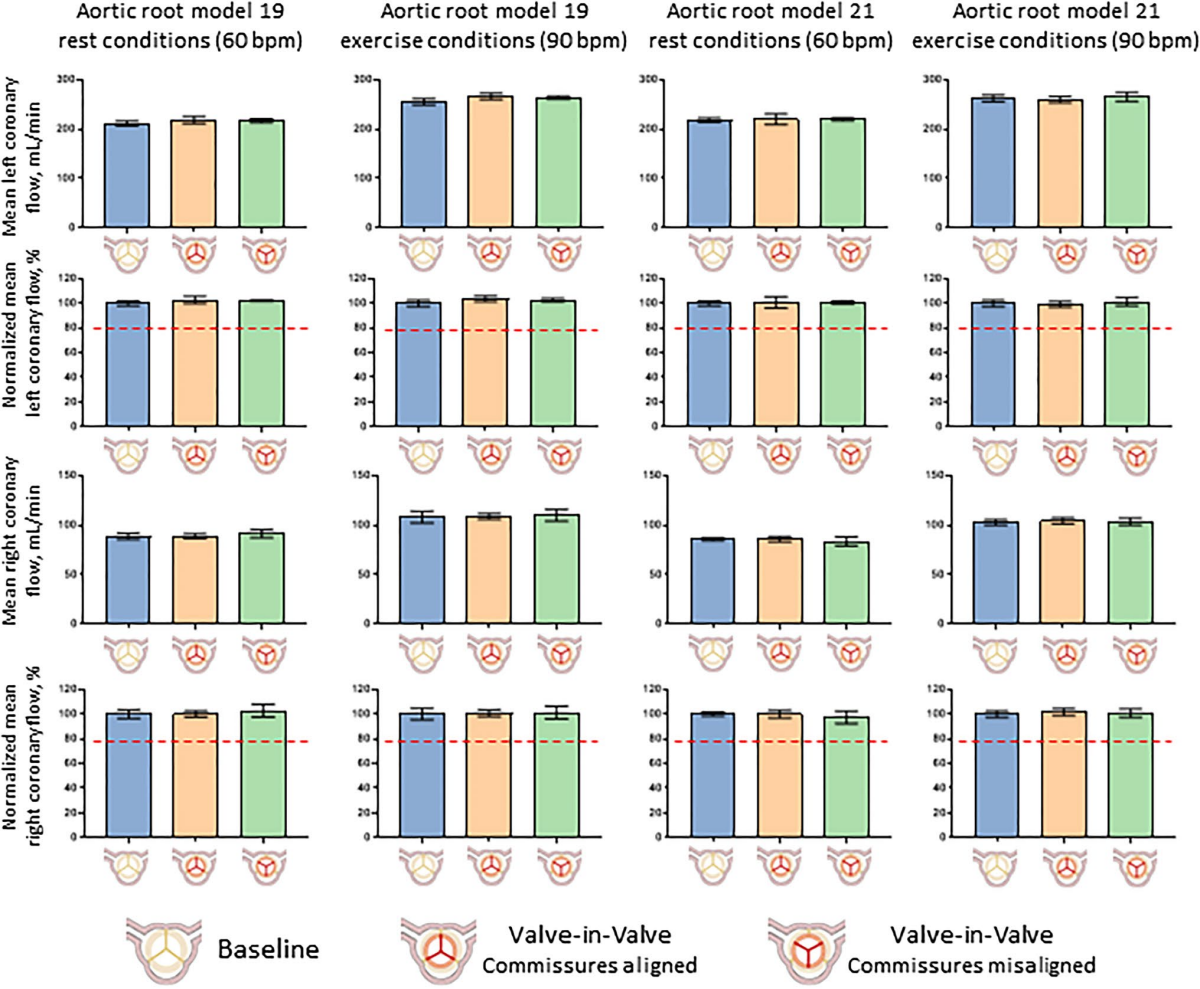
Pre- and post-VIV-TAVI mean left and right coronary flowrates (error bars are 95% confidence intervals) in absolute values (in mL/min) and in percentage values normalized to the baseline mean (in %) for the two aortic root models (model 19 and 21) and for the two loading conditions (rest and exercise). The red dashed lines indicate the cut-off value assumed as relevant impairment of the coronary flow

In all the tested conditions (Fig. 6) the 95% CI lower limits of post-VIV-TAVI left and right normalized coronary flowrates were above the assumed cut-off value for relevant coronary flow impairment (80%). For instance, the mean values of the normalized left coronary flowrate in the aligned commissure configuration at rest loading condition were 102.9% (CI 95%: 99.7 to 106%) and 100.8% (CI 95%: 96.0 to 105.5%) for AR model 19 and 21, respectively, while the mean values of the normalized right coronary flowrate in the aligned commissure configuration at rest loading conditions were 100% (CI 95%: 97.6 to 102.9%) and 100.1% (CI 95%: 96.8 to 103.4%) for AR model 19 and 21, respectively.

## Discussion

Coronary obstruction in VIV-TAVI is a dreadful condition associated with a very high mortality risk. Its mechanism is related to the displacement and fixing of the SAVB leaflets in the proximity of the coronary ostia by the radial forces of the TAVI stent. A narrow AR reduces the area available for the blood flow entering the sinus of Valsalva with potential direct or indirect (such as promoting coagulation state due to low-shear environment in the neo-sinuses [7, 21]) consequences on coronary flow. Another scenario is related to the SAVB leaflets’ adherence to the aortic wall in case the SAVB leaflets extend above the STJ. Indeed, a risk exists that the SAVB leaflets, which are forced to stay open by the TAVI stent, lean towards the aortic wall creating a coated tube potentially excluding the sinuses of Valsalva, and consequently the coronary arteries, from the blood flow [22].

These mechanisms are mainly dependent on the AR geometry and the SAVB characteristics (such as its size, or the fact that the pericardium is mounted internally/externally with respect to the stent) [4]. Furthermore, they might be also influenced by TAVI positioning (implantation depth, commissural alignment [8]) and hemodynamic conditions [23].

Clinically, the complex interplay between these factors limits the possibilities to isolate them and study their separate influence on coronary perfusion alteration in VIV-TAVI. The high mortality risk due to coronary occlusion, however, urges for in-depth understanding for better risk stratification and VIV-TAVI safety improvement. In the literature, 3D printed ARs have been already exploited for procedural pre-planning [24, 25]. Stock et al. and Azadani et al. used aortic sinus Dacron prostheses and human homograft ARs, and investigated the influence of TAVI size, TAVI angular orientation, and coronary ostia height on coronary perfusion post-VIV-TAVI in hemodynamic in vitro tests [26, 27]. In these studies, the AR models were representative of low-risk anatomies.

Our study is the first experimental in vitro work in which the AR geometry was considered a factor potentially affecting the coronary perfusion in VIV-TAVI scenario. The AR computer-aided design allowed us to obtain a versatile paradigmatic model, which potentially can replicate different morphometries by changing the model’s geometric parameters. The 3D printing technology allowed for rapid and high-accuracy manufacturing of the AR models. The pulse duplicator and the coronary flow simulator provided high-controllable physiological-like hemodynamic conditions in terms of pressures and aortic and coronary flow waveforms. All these aspects support the reliability of the obtained results.

Particularly, in this study, the AR model design inputs were defined based on the realistic clinical insights and recreated a high-risk anatomical scenario based on VTCD, SOVD, and SOVH dimensions. Moreover, the smallest SAVB label sizes (19 and 21), with pericardium mounted outside the pivot, were selected to enhance coronary flow impairment risk. The TAVI deployment was controllable in terms of implantation depth and commissural alignment. This in vitro model allowed analyzing coronary flow after VIV-TAVI under rest and exercise simulated conditions.

The results of this study provided no evidence of coronary obstruction or coronary flow disturbance in any of the 72 experimental tests. The chosen AR root anatomies did not trigger any clinically relevant coronary flow alterations post-VIV-TAVI, neither in case of the TAVI commissural misalignment nor under simulated exercise conditions in the analyzed SAVB and TAVI types.

Clinically, the influence of commissural alignment between TAVI and SAVB on the coronary flow is debated. In this in vitro study, the misalignment of the TAVI commissures did not result as a significant factor altering the coronary perfusion. This finding is in line with the experimental results of Azadani et al. [26, 27] and with computational fluid dynamics simulations where the TAVI implantation in a misaligned configuration did not cause differences in the coronary filling with respect to the TAVI implantation in the aligned commissure configuration in 14 patient-specific anatomies [28]. Nonetheless, commissural alignment has been advocated as a key factor to facilitate coronary ostia cannulation; in the misalignment configuration, the TAVI commissures face off coronary ostia, preventing coronary access for future interventions [8].

The used setup allowed us to isolate the effect of hemodynamic conditions on the coronary perfusion (fixed resistances) from the physiological factors that would influence the coronary resistance in the in vivo situation. In this scenario, the change from rest to exercise loading conditions (in terms of heart rate and AoP increase) induced significant increase of coronary flowrate at baseline (in left coronary: + 21%, in right coronary: + 20%) and at VIV-TAVI conditions (in left coronary: + 22%, in right coronary: + 23%). In such controllable experimental conditions, no significant differences between baseline and VIV-TAVI configurations in terms of coronary flowrate parameters were found throughout the same loading condition. Moreover, no relevant changes in coronary perfusion were detected post-VIV-TAVI when compared to the 80% FFR reference cut-off value. The reasonable interpretation is that the local hemodynamics induced by VIV-TAVI complex in the simulated exercise conditions did not change in a way to influence the coronary flow. Of note, the simulated exercise conditions represented a physical activity tolerable by elderly patients, the target population of TAVI. Increasing the heart rate to 160 bpm in the future studies could take into account the expansion of TAVI treatment towards low- and intermediate-risk younger patients’ population [29].

This is a proof-of-concept study, which demonstrated the reliability of the applied in vitro methodology to study the coronary perfusion post-VIV-TAVI, with future application potential. The results should be however carefully translated to clinical practice as the simulated conditions represent a narrow cohort of patients where VTCD was set to 4 mm, i.e., clinical cut-off value for high-risk coronary obstruction, and lower VTCD were not tested. Moreover, in the present study, the AR models had paradigmatic anatomy with symmetric sinuses of Valsalva and centered coronary ostia. The applied methodologies however permit AR geometry variations, including, e.g., SAVB angular inclination with respect to the AR axis, or replicating patient-specific AR anatomies, or housing other types of SAVB and TAVI prostheses. The AR models used in this study were rigid, lacking the compliance of the natural AR. This allowed accurate AR models’ geometry reproduction which was also independent from the hemodynamic conditions. Normally, in VIV-TAVI, the SAVB is degenerated, and calcifications present on the leaflets could play a role on coronary perfusion after VIV-TAVI. The SABV used in this study were not degenerated, as calcification simulation would be uncontrollable, and would introduce additional confounding factors (calcification location, dimensions, mechanical properties). In the future, experimental SAVB degeneration strategies could be employed [30]. In this study, thrombosis-induced coronary flow impairment was not studied as blood was not used.

## Conclusions

Our in vitro study does not provide any evidence for coronary obstruction or coronary flow impairment during VIV-TAVI in a small aortic root configuration with TAVI commissural aligned or misaligned configuration, neither in rest nor in exercise conditions. The data highlight that test setup represents a high-fidelity reproducible tool for coronary flow analysis. The versatility of our setup allows to replicate multiple clinical scenarios. The setup will be further employed for in-depth studies of the coronary flow alteration phenomenon in VIV-TAVI, potentially improving our knowledge and providing possible insights for better risk stratification, towards a better patients’ safety.

## Supplementary Information

Below is the link to the electronic supplementary material.

Supplementary file1 Endoscopic en face videos of tested prostheses housed in an aortic root model of geometry considered risky for coronary occlusion at the baseline (Trifecta GT 19) and post valve-in-valve TAVI treatment (Trifecta GT 19 + Portico 23) with commissural alignment and misalignment configuration. (MP4 82384 KB)

## Data Availability

The datasets generated during and/or analysed during the current study are available from the corresponding author on reasonable request.

## Abbreviations

VIV-TAVI: Transcatheter aortic valve-in-valve implantation
SAVB: Surgical aortic valve bioprosthesis
TAVI: Transcatheter aortic valve replacement
AR: Aortic root
STJ: Sinotubular junction
SOVD: Sinus of Valsalva diameter
AD: Annulus diameter
VTCD: Valve-to-coronary distance
CO: Cardiac output
SV: Stroke volume
BV: Backflow volume
AoP: Mean aortic pressure
